# Stroke in Athletes with Atrial Fibrillation: A Narrative Review

**DOI:** 10.3390/diagnostics15010009

**Published:** 2024-12-25

**Authors:** Joana Certo Pereira, Maria Rita Lima, Francisco Moscoso Costa, Daniel A. Gomes, Sérgio Maltês, Gonçalo Cunha, Hélder Dores, Pedro Adragão

**Affiliations:** 1 Hospital de Santa Cruz, 2790-134 Lisbon, Portugal; mlima@ulslo.min-saude.pt (M.R.L.); fmoscosocosta@gmail.com (F.M.C.); danielssagomes@gmail.com (D.A.G.); smaltes@ulslo.min-saude.pt (S.M.); gcunha@ulslo.min-saude.pt (G.C.); padragao@ulslo.min-saude.pt (P.A.); 2 Hospital da Luz, 1500-650 Lisbon, Portugal; heldores@hotmail.com; 3 CHRC, Comprehensive Health Research Center, 1600-560 Lisbon, Portugal; 4 Associate Laboratory REAL (LA-REAL), 1099-085 Lisbon, Portugal; 5 NOVA Medical School, NOVA University Lisbon, 1169-056 Lisbon, Portugal; 6 CoLab TRIALS, 7002-554 Évora, Portugal

**Keywords:** athletes, stroke, atrial fibrillation

## Abstract

Atrial fibrillation (AF) is the most common sustained arrhythmia, linked with a significantly heightened risk of stroke. While moderate exercise reduces AF risk, high-level endurance athletes paradoxically exhibit a higher incidence. However, their stroke risk remains uncertain due to their younger age, higher cardiovascular fitness, and lower rate of comorbidities. Several key studies highlight that AF may increase the risk of stroke in endurance athletes, particularly those over 65. However, the overall risk within this population remains relatively low. Notably, older male athletes show a higher AF incidence but experience lower stroke risk than their non-athletic counterparts. Regular physical activity prior to a first stroke appears to reduce mortality, though recurrent stroke risk in athletes with AF mirrors that of non-athletes, despite an elevated AF incidence. Management of AF in athletes is complex, with limited evidence guiding anti-thrombotic strategies. In this setting, specific recommendations are sparse, particularly in sports where bleeding risk is heightened. Individualized management, emphasizing shared decision-making, is critical to balance stroke prevention with athletic performance. Rhythm control strategies, such as catheter ablation, may be a reasonable first-line treatment option for athletes, particularly in those desiring to avoid long-term medication. This review synthesizes the current literature on the incidence, predictors, and management of stroke in athletes with AF.

## 1. Introduction

Atrial fibrillation (AF) remains the most common cardiac arrhythmia, affecting millions of individuals worldwide [1]. Due to its potential for systemic embolism, AF significantly raises the risk of stroke, being responsible for approximately one-third of ischemic strokes [2]. While AF customarily occurs in older populations and in those with significant comorbidities, its occurrence in younger and healthy individuals, including athletes, has increasingly attracted attention in the past years [3,4]. Current data suggest a dichotomous “J or U-shaped” relationship between exercise and the incidence of AF. Moderate physical activity confers cardiovascular (CV) benefits and lowers AF incidence, while intensive and prolonged endurance exercise may increase the risk of AF [5]. Despite the increasing recognition of AF in athletes, it remains unclear whether exercise-induced AF is associated with an elevated stroke risk. In these individuals, the risk profile may differ from that of the general population due to their younger age, enhanced CV fitness, and possible use of performance-enhancing drugs. Notably, evidence suggests that a significant proportion of athletes are entirely free of risk factors, while only a small minority present high CV risk [6,7]. Moreover, traditional risk screening scores (e.g., CHA2DS2-VASc, or CHA2DS2-VA) have not been extensively validated among these cohorts.

Understanding stroke risk in athletes with AF is essential for devising treatment strategies that balance effective stroke prevention and AF management with a minimal impact on performance. The aim of this paper was to perform a narrative review of the current knowledge regarding the incidence, predictors, clinical impact, and management of stroke in athletes with AF.

## 2. Materials and Methods

A literature search of medical electronic databases through August 2024 was performed to identify studies examining the relationship between AF and stroke in athletes. Search words included, but were not restricted to ‘athlete’, ‘atrial fibrillation’, ‘stroke’, and ‘embolism’. Any study that explored the relationship between stroke and athletes with AF was considered for inclusion.

## 3. Relationship Between Exercise and Atrial Fibrillation

Athletes have an approximately two-to-five-fold increased lifetime risk of AF compared to sedentary individuals, despite having a lower prevalence of conventional AF risk factors [8,9]. Specific risk factors for AF in athletes include male sex, tall stature, a total lifetime exercise dose exceeding 1500–2000 h, and participation in endurance sports, particularly running, cycling, and cross-country skiing [10,11,12]. Interestingly, in contrast to endurance sports, an increased risk of AF has not been demonstrated in strength-based sports. This may be due to the greater hemodynamic stress typically associated with endurance activities over prolonged periods [13,14]. Nevertheless, mixed-sports may also portray an increased risk of developing AF; however, the wide variety of activities included in such exercises make this finding difficult to interpret [9].

The CARDIO-FIT study demonstrated that patients who achieved significant improvements in aerobic capacity also experienced overall improvements in classical risk factors, along with enhancements in left ventricular and left atrial size and diastolic function, providing substantial benefits for those with AF [15]. Despite AF prevalence generally increasing with age, younger athletes still have a higher incidence when compared to nonathletes. A meta-analysis by Newman et al. [9] reported that younger athletes have a significantly higher relative risk of AF compared to older athletes (OR: 3.60; 95% CI 2.09 to 6.29 vs. OR: 1.76; 95% CI 0.97 to 3.21), a finding consistent with the results of Ayinde et al. [16]. Notably, the same meta-analysis reported an OR of 1.76 for older athletes, which is still significantly higher than in non-athletes, highlighting the importance of assessing AF risk across all age groups. The increased risk in younger athletes has been attributed to adrenergic surges during exercise, as described by Hoogsteen et al., while older athletes are more prone to vagally induced AF after exercise, often linked to secondary autonomic dysfunction. The relatively lower association of AF in older athletes compared to younger athletes may be explained by the age-related increase in AF risk, which occurs independently of physical activity levels [17]. Although this association was initially observed in males, recent studies suggest the same association for female endurance athletes [18,19,20,21].

While endurance exercise clearly raises the risk of AF in men, the prevalence of AF among athletes remains low [22]. Additionally, resistance exercise continues to offer a notable cardiovascular and overall mortality benefit that outweighs the increased AF risk. Therefore, the heightened AF risk should be balanced against the considerable health benefits it provides.

## 4. Pathophysiology of Atrial Fibrillation in Athletes

Coumel’s triangle [23], which includes the arrhythmogenic substrate, modulating factors, and triggers, is key to understanding AF pathophysiology. Rebecchi and colleagues recently introduced the concept of the “Autonomic Coumel’s Triangle”, emphasizing the autonomic nervous system (ANS)’s influence on both triggers and substrates in AF [24].

The ANS can exacerbate ectopic focus through sympathetic and parasympathetic activation and shortening of the atrial refractory period via the parasympathetic system. The role of the ANS is crucial in promoting AF in athletes, and its influence may vary according to the type and intensity of exercise [25]. Endurance exercise increases parasympathetic activity at rest, while adrenergically mediated AF can occur during intense exercise due to high sympathetic tone. Athletes with a greater volume of exercise have also a higher burden of premature atrial contractions compared to less-trained or sedentary individuals. All these factors together may facilitate inducibility and AF maintenance in this population [26,27,28]. Despite its relevance, the ANS does not appear to be the sole factor contributing to the pathogenesis of exercise-induced AF. Well-documented physiological structural changes in athletes, referred as athlete’s heart, include left and right ventricular geometry adaptations, such as hypertrophy and dilation, as well as left and right atrial dilation and myocardial strain decrease [29]. These changes, particularly the left atrial enlargement and fibrosis, modify the physical and electrical atrial wall’s properties, further perpetuating AF [29,30]. This type of atrial myopathy resulting from exercise-induced hemodynamic stretch may be part of the substrate for AF in athletes. This is particularly striking as, in contrast to non-athlete AF patients, left ventricular (LV) diastolic function is usually preserved [31,32,33,34]. Furthermore, increasing evidence links inflammation to the onset and persistence of AF, driving structural and electrophysiological changes that contribute to atrial remodelling [35]. Electrical remodelling promotes micro-re-entry lead by the shortening the atrial refractory period and increased conductivity, with oxidative stress exacerbating these effects through calcium interactions [36].

In endurance athletes, it is well-established that intense and prolonged exercise can induce a generalized pro-inflammatory state, depending on the exercise’s duration and intensity, particularly when not balanced with adequate recovery periods [37,38,39,40].

## 5. Stroke in Athletes with Atrial Fibrillation

Stroke represents a serious complication of AF, irrespective of whether it manifests as paroxysmal, persistent, or permanent [41,42]. Approximately 25% of ischemic strokes have a cardioembolic origin, with AF being the leading cause [43]. AF not only increases the risk of stroke, but these cardioembolic events also tend to have higher severity and mortality [44,45].

The relationship between AF and stroke is complex. One proposed mechanism is impaired atrial contractility leading to blood stasis and increased thromboembolism risk [46]. Further atrial abnormalities, such as endothelial dysfunction, fibrosis, impaired myocyte function, chamber dilatation, and mechanical dysfunction of the left atrial appear to be linked to stroke, suggesting that AF may be a secondary marker of underlying atrial pathology [47]. Additionally, AF is often accompanied by other comorbidities independently associated with an increased stroke risk, including hypertension, diabetes, heart failure, dyslipidaemia, sleep apnoea, tobacco use, and obesity [48,49,50]. Nevertheless, CV risk factors may scarcely occur in athletes, possibly suggesting a lower contribution of these factors to stroke risk in athletes [6].

Recently, overall vascular calcification has been linked to an increased risk of AF, adverse cardiovascular events, and worse stroke outcomes [51]. Indeed, in the MESA cohort, coronary artery calcification (CAC) was associated with a higher risk of AF, particularly in younger participants, with the risk increasing alongside CAC progression [52]. Additionally, in this population, CAC was strongly linked to the 10-year risk of death from coronary heart disease, non-fatal MI, and both fatal and non-fatal stroke [53]. Two other studies have also reported similar findings [54,55]. Against this background, artery calcification may serve as a novel risk marker for AF and stroke [56]. Athletes, despite presenting a lower prevalence of CV risk factors, have been shown to present unexpectedly high levels of CAC, particularly in veteran athletes engaged in lifelong exercise [57,58,59]. However, the relationship between CAC and the risk of AF and stroke in athletes is not yet well established, presenting a possible area for further research.

In non-athletes, nonvalvular AF is well-documented to increase stroke risk by fivefold, while AF associated with mitral stenosis raises stroke risk by twenty times [60]. However, the exact stroke risk related to AF in athletes remains unclear, as few studies have assessed this question prospectively. Table 1 provides a summary of the current evidence regarding stroke in athletes. In 2020, Myrstad et al. reported on the association between AF and stroke in athletes [61]. The study, involving 2626 men and women who participated in the 1999 Birkebeinerrennet, a 54 km cross-country ski race, examined the incidence of self-reported AF, stroke, and related conditions. The relative risk of stroke was 85% higher in athletes with AF (OR 1.85, 95% CI 0.99–3.46, *p* = 0.06). Both self-reported and adjudicated AF were associated with a higher prevalence of stroke in patients aged 65 and older (OR 2.16, 95% CI 1.07–4.38, *p* < 0.05 vs. OR 2.38, 95% CI 1.05–5.40, *p* < 0.05), but not in those younger than 65.

Additionally, the AFLETES study [62] aimed to assess the risk of stroke in veteran endurance athletes with AF through an international online survey involving 942 athletes over the age of 40. In the study cohort, 20% of participants reported AF, and 3% had experienced a stroke, of which 54% also had AF. Multivariable analysis revealed that lifetime exercise dose (OR 1.02, 95% CI 1.00–1.03, *p* = 0.02) and swimming (OR 1.56, 95% CI 1.02–2.39, *p* = 0.04) were associated with AF. Furthermore, AF was linked to a fourfold increased risk of stroke (OR 4.18, 95% CI 1.80–9.72, *p* < 0.01), even in individuals with a low CHA2DS2-VASc score (OR 4.20, 95% CI 1.83–9.66, *p* < 0.01). These findings suggest that endurance athletes with AF may be at an elevated risk of stroke, regardless of traditional risk factors.

Nonetheless, the recent Tromsø Study [18], suggested that the risk of stroke associated with exercise-related AF might be lower compared to the overall AF population. This study followed 505 male athletes with a median age of 68 who regularly participated in a long-distance ski race over a median period of 14 years, comparing them to 1867 men of the same age from the general population. Outcomes were self-reported AF and stroke. Moreover, the cohort reported a median of 36 years of regular endurance training, with 90% engaging in moderate or vigorous physical activity in the past year. The results showed that athletes had a higher prevalence AF prevalence (28.5% vs. 17.8%, risk ratio 1.88), but a lower stroke risk (5.4% vs. 9.7%, risk ratio of 0.60) compared to non-athletes. Compared to athletes without AF, athletes with AF had a twofold increase in stroke risk, while non-athletes with AF experienced a nearly fourfold increase in stroke risk. Additionally, Svedberg et al. previously investigated the relationship between endurance training, AF, and stroke in 208,654 Swedish cross-country skiers (both male and female) who participated in the Vasaloppet from 1989 to 2011, comparing them to a matched cohort of 527,448 non-skiers [63]. Both male and female skiers had lower stroke incidence than non-skiers (HR 0.64). Skiers with AF had a higher stroke incidence than those without AF, but after an AF diagnosis, they had lower stroke incidence (HR 0.73) and mortality (HR 0.57) compared to non-skiers with AF.

Ulf Hallmarker and colleagues [64] also examined the relationship between AF and stroke in long-distance ski race participants who were diagnosed with a first stroke event. The study included participants from the Vasaloppet and matched controls (n = 708,604) between 1994 and 2010, during which time 5964 patients were hospitalized for a first stroke. In this cohort, skiers had a lower stroke hospitalization rate compared to non-skiers (0.5% vs. 1%) as well as a lower risk of recurrent stroke or death (HR 0.76). After adjusting for smoking and socioeconomic factors, the reduction in death risk remained significant (HR 0.70), though not for recurrent stroke. There was a higher AF incidence in skiers than non-skiers (20.4% vs. 14.7%) at the first stroke, but skiers had a lower recurrent stroke rate (4.8% vs. 6%). Death and recurrent stroke rates were higher in individuals with AF compared to those without AF, in both skiers and non-skiers.

**Figure 1:**
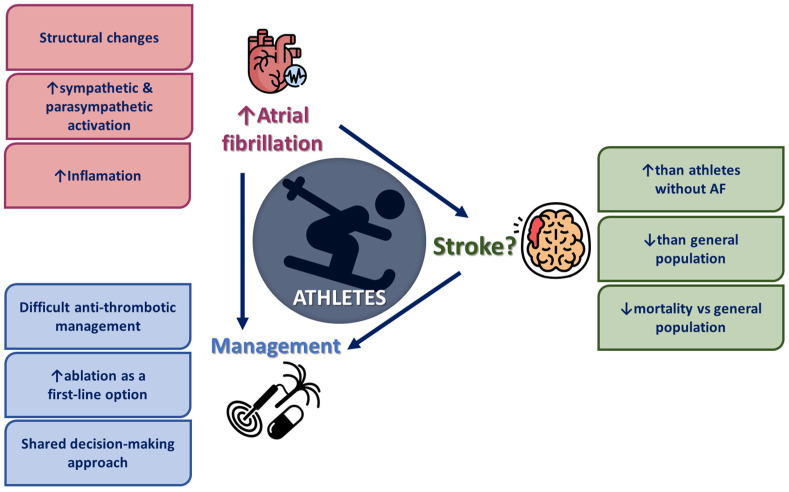
Relationship between exercise, AF, and stroke.

Overall, the data suggest that AF may increase stroke risk in endurance athletes, particularly in those aged 65 and older. However, while AF raises individual stroke risk, absolute population-level risk remains low. Older male athletes face a higher risk of AF but a lower stroke risk compared to matched non-athletes. Additionally, regular physical activity before a first stroke may reduce mortality, though the risk of recurrent stroke remains similar to non-athletes despite higher AF rates. Figure 1 summarizes the complex relationship between physical activity and AF, stroke, and management options.

## 6. Management of Athletes with Atrial Fibrillation

Data are scarce regarding the optimal management of AF in athletes. Thus, decisions regarding treatment strategies are largely based on extrapolating data from clinical trials involving non-athletes and expert opinions. Recent 2024 guidelines recommend patient-centred care for all AF patients, following AF-CARE principles: [C] comorbidity and risk factor management; [A] avoid stroke and thromboembolism; [R] reduce symptoms by rate and rhythm control; [E] evaluation and dynamic reassessment [65]. In line with these recommendations, the “2024 Heart Rhythm Society (HRS) Expert Consensus Statement on Arrhythmias in the Athlete” suggests a management strategy that includes risk factor management, thromboembolism prevention, and rhythm control [66]. Standard risk factors should be addressed and modified, similarly to non-athletes. While data on detraining are limited, it may be effective for some individuals and can be considered as an option. However, complete detraining is generally discouraged, as a sedentary lifestyle is more strongly associated with a higher prevalence of AF [15]. The use of performance-enhancing drugs is an athlete-specific risk factor, with some studies suggesting possible associations between certain substances, like creatine, anabolic steroids, and AF [22,67,68,69].

Although AF is not inherently a contraindication for exercise, intensive sports participation should be restricted in athletes with frequent, symptomatic AF or high-risk underlying conditions until effective management is achieved [66]. Furthermore, a comprehensive evaluation is essential to exclude underlying conditions, such as inherited arrhythmia syndromes, cardiomyopathies, or congenital heart disease, which may increase the risk of sudden cardiac arrest and potentially contraindicate sports participation [70,71].

Regarding thromboembolism prevention, risk scores, like CHA2DS2-VASc and the newly introduced CHA2DS2-VA [65], have not been validated in athletes, and international recommendations suggest considering anticoagulant therapy based on thromboembolism risk, similar to the general population [66,72,73]. There is heightened concern about the bleeding risk in athletes, particularly those involved in sports with a risk of trauma (e.g., contact sports, such as football, rugby, wrestling, martial arts, and boxing) [74,75]. For this reason, the 2020 European guidelines recommend that sports involving direct physical contact or a risk of trauma should be avoided in patients on direct oral anticoagulants [72]. However, excluding athletes from competitions could potentially end their careers, resulting in significant psychological and financial consequences. Some studies have proposed short-term oral anticoagulation (OAC) discontinuation strategies around competition timing, aiming to minimize hemorrhagic risk without significantly increasing thrombotic risk by resuming anticoagulation shortly after competition [76,77]. Small pilot studies have suggested the feasibility of this “pill-in-the-pocket” anticoagulation strategy for low-stroke-risk AF patients, but limited data prevent conclusions on long-term stroke outcomes [78,79].

Given the absence of concrete evidence to guide anticoagulation management in athletes, the Heart Rhythm Society 2024 Expert Consensus recommends that decisions on sport participation and anticoagulation management should be individually tailored and guided by a shared decision-making process, considering patient preferences, stroke risk, and the type of sport [66]. Alternative strategies, such as left atrial appendage occlusion may also be considered in a minority of high-risk athletes unable to undergo oral anticoagulation [66,80]. Safer anticoagulant alternatives are needed for young athletes with AF who wish to continue practicing contact sports, allowing them to continue their active lifestyles without jeopardizing their safety.

Therapeutic rate control in athletes is challenging, and while most anti-arrhythmic drugs are not absolute contraindications for sport resumption in athletes with AF, their use necessitates careful evaluation and monitoring.

Beta blockers and non-dihydropyridine calcium channel blockers may impair performance, with beta blockers also prohibited in some sports (e.g., archery, shooting) under World Anti-Doping Agency regulations. Their poor tolerability due to negative effects on performance, combined with the relative ineffectiveness of calcium channel blockers for exertional AF, limits their feasibility as therapeutic options for athletes [81,82]. Rhythm control is generally preferred, with flecainide and propafenone being effective options for managing paroxysmal AF. However, these class IC agents carry a risk of proarrhythmia, requiring coadministration with AV nodal blockers, a 48 h hiatus from sports to allow drug clearance, and stress testing to monitor for QRS widening. Class III agents (e.g., sotalol) may impact performance and pose risks in dehydrated athletes, while amiodarone is relatively contraindicated due to toxicities. Thorough assessment and monitoring are essential before returning to sport [66].

AF catheter ablation is recommended for those unresponsive to medical therapy and is often preferred by athletes in order to avert drug therapy [83]. Several studies emphasize the benefits of early rhythm control in a general population. The EAST-AFNET 4 trial [84] found that early rhythm control significantly reduced CV death, stroke, and hospitalization compared to usual care. Similarly, the RAFAS trial [85] showed that early rhythm control after an acute ischemic stroke lowered sustained AF and recurrent stroke within 12 months, without increasing adverse outcomes. A subsequent meta-analysis found that early rhythm therapy was associated with a lower risk of all-cause mortality, cardiovascular mortality, stroke, and heart failure hospitalization compared to rate control. Specifically, in terms of stroke risk, six studies showed that early rhythm control significantly reduced the risk of ischemic stroke (RR 0.77, 95% CI 0.67–0.87, *p* < 0.001, I^2^ = 64%) [86]. Moreover, when compared to antiarrhythmic drugs, catheter ablation appears to be superior in preventing AF recurrence and improving quality of life [87,88,89,90]. A Swedish study showed that it was associated with a reduction in all-cause mortality and stroke compared to medical therapy. The decrease in all-cause mortality was particularly notable, with stroke reduction showing a trend favoring catheter ablation [91]. However, although observational data indicate a potential reduction in stroke incidence among patients undergoing AF ablation, randomized controlled trials are still needed to validate this benefit.

The current European guidelines recommend catheter ablation as a reasonable first-line option as part of a rhythm control strategy in patients with paroxysmal AF, to reduce the symptoms, recurrence, and progression of AF. The similar efficacy of AF catheter ablation in both the athletic and non-athletic populations has been shown in a recent meta-analysis of 9 observational studies involving 1129 participants, 51% of whom were endurance athletes. It found no significant difference in atrial arrhythmia recurrence rates between endurance athletes and non-endurance athletes following catheter ablation (RR 1.04, *p* = 0.54) [92]. These findings suggest that AF catheter ablation may be equally effective and safe in athletes, supporting its use as a possible first-line treatment option, particularly in those wishing to avoid drug therapy. However, further research is required to establish the benefit of catheter ablation in these patients.

## 7. Conclusions

Despite being higher when compared to the general population, AF incidence in high-endurance athletes remains relatively low and appears to confer a small risk of stroke. Nevertheless, for the associated embolic risk, specific recommendations on the optimal anti-thrombotic strategy in athletes are currently lacking, particularly when considering the risk of bleeding events related to certain sports. Rhythm control with anti-arrhythmic drugs and/or catheter ablation should be pursued, with ablation being a possible first-line option in athletes, especially in those wishing to avoid drug therapy. Given the lack of evidence to guide AF management in athletes, treatment must be patient-tailored and guided by a shared decision-making approach.

## Figures and Tables

**Table 1:** Current evidence regarding stroke in athletes.

Title	Objective and Methods	Population	Main Results
Stroke in endurance athletes with atrial fibrillation [61]	To study the association between AF and stroke in athletes. Self-reported AF and stroke	N = 2626, Athletes ≥ 40 years Cross-country skiers Mean age (years): 66.5 (AF) and 64 (no-AF) CHA2DS2-VASc-score ≥ 2: 36.3% (AF) and 26.3% (no-AF)	Stroke prevalence: 6.8% in athletes with AF. 3.9% in athletes without AF. AF was linked to higher stroke rates in athletes 65 years and older.
The AFLETES Study: Atrial Fibrillation in Veteran Athletes and the Risk of Stroke [62]	To investigate risk of stroke in veteran endurance athletes who develop AF (vs. sinus rhythm) Self-reported AF and stroke	N = 942, Athletes ≥ 40 years ++ Cycling (72%), running (59%), and triathlon (26%). (Average 9 h/week, 20 years) 84% male, 96% Caucasian Mean age (years): 56.6 (AF) vs. 51.3 (no-AF) CHA2DS2-VASc-score ≥ 2: 6.2%	Stroke prevalence: 7% in athletes with AF. 2% in athletes without AF. AF was independently associated with stroke in athletes, even in those with a low CHA2DS2-VASc score.
Birkebeiner Ageing Study and the Tromsø Study [18]	To investigate AF and stroke risk in older athletes exposed to prolonged endurance training Self-reported AF and stroke (at baseline or 10-year follow-up)	N = 2372, Skiers and a matched group from general population, ≥65 years (505 male athletes; 1867 non-athletes) Cross-country skiers (median: 14 years). Median age (years): 68 (athletes) and 70 (non-athletes)	Stroke prevalence: 8.3% in athletes with AF. 14.2% in non-athletes with AF. Despite a higher risk of AF, athletes with AF may have a lower risk of stroke than non-athletes with AF.
Long-Term Incidence of Atrial Fibrillation and Stroke Among Cross-Country Skiers [63]	To investigate the links between endurance training, atrial fibrillation, stroke, and sex differences. Followed until first event of AF or stroke	N = 736,102, Skiers + matched group of non-skiers (208,654 athletes; 527,448 non-athletes) Cross-country skiers Mean age (years): 37.3 (athletes) and 42.1 (non-athletes) Mean CHA2DS2-VASc-score: 0.96 in athletes with AF and 1.38 in non-athletes with AF.	Stroke prevalence: 7.6% in athletes with AF. 9.7% in non-athletes with AF. 0.6% in athletes without AF. 1.2% in non-athletes without AF. Athletes with atrial fibrillation had a lower incidence of stroke and lower mortality compared with non-athletes with atrial fibrillation
Risk of Recurrent Stroke and Death After First Stroke in Long-Distance Ski Race Participants [64]	To study stroke recurrence, death, and AF in highly active individuals after a first stroke compared to the general population. Followed from the diagnosis of stroke.	N = 5964, Patients hospitalized with a first-time stroke (1083 athletes; 4881 non-athletes) Cross-country skiers Mean age (years): 64 (both groups)	Death: 3.8% in athletes 5.8% in non-athletes Re-Stroke: 4.8% in athletes 6.0% in non-athletes Athletes with a stroke have a lower risk of death, while their risk for recurrent stroke is similar to that of non-athletes.

## Data Availability

No new data were created or analyzed in this study. Data sharing is not applicable to this article.

## References

[1] Chugh S.S., Roth G.A., Gillum R.F., Mensah G.A. (2014). Global Burden of Atrial Fibrillation in Developed and Developing Nations. Glob. Heart.

[2] Friberg L., Rosenqvist M., Lindgren A., Terént A., Norrving B., Asplund K. (2014). High Prevalence of Atrial Fibrillation Among Patients with Ischemic Stroke. Stroke.

[3] Benjamin E.J., Muntner P., Alonso A., Bittencourt M.S., Callaway C.W., Carson A.P., Chamberlain A.M., Chang A.R., Cheng S., Das S.R. (2019). Heart Disease and Stroke Statistics—2019 Update: A Report From the American Heart Association. Circulation.

[4] Allan V., Honarbakhsh S., Casas J.-P., Wallace J., Hunter R., Schilling R., Perel P., Morley K., Banerjee A., Hemingway H. (2017). Are cardiovascular risk factors also associated with the incidence of atrial fibrillation?. Thromb. Haemost..

[5] Morseth B., Løchen M.L., Ariansen I., Myrstad M., Thelle D.S. (2018). The ambiguity of physical activity, exercise and atrial fibrillation. Eur. J. Prev. Cardiol..

[6] D’ascenzi F., Caselli S., Alvino F., Digiacinto B., Lemme E., Piepoli M., Pelliccia A. (2019). Cardiovascular risk profile in Olympic athletes: An unexpected and underestimated risk scenario. Br. J. Sports Med..

[7] Carlsson S., Olsson L., Farahmand B.Y., Hållmarker U., Ahlbom A. (2007). Skiers in the long-distance ski race invest in their health. Lakartidningen.

[8] Abdulla J., Nielsen J.R. (2009). Is the risk of atrial fibrillation higher in athletes than in the general population? A systematic review and meta-analysis. Europace.

[9] Newman W., Parry-Williams G., Wiles J., Edwards J., Hulbert S., Kipourou K., Papadakis M., Sharma R., O’Driscoll J. (2021). Risk of atrial fibrillation in athletes: A systematic review and meta-analysis. Br. J. Sports Med..

[10] Calvo N., Ramos P., Montserrat S., Guasch E., Coll-Vinent B., Domenech M., Bisbal F., Hevia S., Vidorreta S., Borras R. (2016). Emerging risk factors and the dose–response relationship between physical activity and lone atrial fibrillation: A prospective case–control study. Europace.

[11] Crump C., Sundquist J., Winkleby M.A., Sundquist K. (2018). Height, Weight, and Aerobic Fitness Level in Relation to the Risk of Atrial Fibrillation. Am. J. Epidemiol..

[12] Molina L., Mont L., Marrugat J., Berruezo A., Brugada J., Bruguera J., Rebato C., Elosua R. (2008). Long-term endurance sport practice increases the incidence of lone atrial fibrillation in men: A follow-up study. EP Eur..

[13] Flannery M.D., Kalman J.M., Sanders P., La Gerche A. (2017). State of the Art Review: Atrial Fibrillation in Athletes. Heart Lung Circ..

[14] Opondo M.A., Aiad N., Cain M.A., Sarma S., Howden E., Stoller D.A., Ng J., van Rijckevorsel P., Hieda M., Tarumi T. (2018). Does High-Intensity Endurance Training Increase the Risk of Atrial Fibrillation?. Circ. Arrhythm. Electrophysiol..

[15] Pathak R.K., Elliott A., Middeldorp M.E., Meredith M., Mehta A.B., Mahajan R., Hendriks J.M., Twomey D., Kalman J.M., Abhayaratna W.P. (2015). Impact of CARDIOrespiratory FITness on Arrhythmia Recurrence in Obese Individuals with Atrial Fibrillation. J. Am. Coll. Cardiol..

[16] Ayinde H., Schweizer M.L., Crabb V., Ayinde A., Abugroun A., Hopson J. (2018). Age Modifies the Risk of Atrial Fibrillation among Athletes: A Systematic Literature Review and Meta-Analysis. IJC Heart Vasc..

[17] Brunetti N.D., Santoro F., Correale M., De Gennaro L., Conte G., Di Biase M. (2016). Incidence of Atrial Fibrillation Is Associated with Age and Gender in Subjects Practicing Physical Exercise: A Meta-Analysis and Meta-Regression Analysis. Int. J. Cardiol..

[18] Johansen K.R., Ranhoff A.H., Sørensen E., Nes B.M., Heitmann K.A., Apelland T., Sandbakk S.B., Wilsgaard T., Løchen M.-L., Thelle D.S. (2022). Risk of atrial fibrillation and stroke among older men exposed to prolonged endurance sport practice: A 10-year follow-up. The Birkebeiner Ageing Study and the Tromsø Study. Open Heart.

[19] Drca N., Larsson S.C., Grannas D., Jensen-Urstad M. (2023). Elite female endurance athletes are at increased risk of atrial fibrillation compared to the general population: A matched cohort study. Br. J. Sports Med..

[20] Claessen G., Colyn E., La Gerche A., Koopman P., Alzand B., Garweg C., Willems R., Nuyens D., Heidbuchel H. (2011). Long-term endurance sport is a risk factor for development of lone atrial flutter. Heart.

[21] Myrstad M., Johansen K.R., Sørensen E., Løchen M.L., Ranhoff A.H., Morseth B. (2024). Atrial fibrillation in female endurance athletes. Eur. J. Prev. Cardiol..

[22] Kammer R.T. (2005). Lone Atrial Fibrillation Associated with Creatine Monohydrate Supplementation. Pharmacotherapy.

[23] Coumel P. (1993). Cardiac Arrhythmias and the Autonomic Nervous System. J. Cardiovasc. Electrophysiol..

[24] Rebecchi M., Fanisio F., Rizzi F., Politano A., De Ruvo E., Crescenzi C., Panattoni G., Squeglia M., Martino A., Sasso S. (2023). The Autonomic Coumel Triangle: A New Way to Define the Fascinating Relationship between Atrial Fibrillation and the Autonomic Nervous System. Life.

[25] Sanchis-Gomar F., Perez-Quilis C., Lippi G., Cervellin G., Leischik R., Löllgen H., Serrano-Ostáriz E., Lucia A. (2017). Atrial fibrillation in highly trained endurance athletes—Description of a syndrome. Int. J. Cardiol..

[26] Wilhelm M., Roten L., Tanner H., Wilhelm I., Schmid J.P., Saner H. (2011). Atrial Remodeling, Autonomic Tone, and Lifetime Training Hours in Nonelite Athletes. Am. J. Cardiol..

[27] Boraita A., Heras M.-E., Valenzuela P.L., Diaz-Gonzalez L., Morales-Acuna F., Alcocer-Ayuga M., Bartolomé-Mateos S., Santos-Lozano A., Lucia A. (2022). Holter-determined arrhythmias in young elite athletes with suspected risk: Insights from a 20-year experience. Front. Cardiovasc. Med..

[28] Bjørnstad H., Storstein L., Dyre Meen H., Hals O. (1994). Ambulatory Electrocardiographic Findings in Top Athletes, Athletic Students and Control Subjects. Cardiology.

[29] Burstein B., Nattel S. (2008). Atrial Fibrosis: Mechanisms and Clinical Relevance in Atrial Fibrillation. J. Am. Coll. Cardiol..

[30] Schotten U., Verheule S., Kirchhof P., Goette A. (2011). Pathophysiological Mechanisms of Atrial Fibrillation: A Translational Appraisal. Physiol. Rev..

[31] Sørensen E., Myrstad M., Solberg M.G., Øie E., Platonov P.G., Carlson J., Tveit A., Aarønæs M. (2023). Left atrial dyssynchrony in veteran endurance athletes with and without paroxysmal atrial fibrillation. Echocardiography.

[32] Trivedi S.J., Claessen G., Stefani L., Flannery M.D., Brown P., Janssens K., Elliott A., Sanders P., Kalman J., Heidbuchel H. (2020). Differing mechanisms of atrial fibrillation in athletes and non-athletes: Alterations in atrial structure and function. Eur. Heart J. Cardiovasc. Imaging.

[33] Sørensen E., Myrstad M., Solberg M.G., Øie E., Tveit A., Aarønæs M. (2021). Left atrial function in male veteran endurance athletes with paroxysmal atrial fibrillation. Eur. Heart J. Cardiovasc. Imaging.

[34] Mont L., Tamborero D., Elosua R., Molina I., Coll-Vinent B., Sitges M., Vidal B., Scalise A., Tejeira A., Berruezo A. (2008). Physical activity, height, and left atrial size are independent risk factors for lone atrial fibrillation in middle-aged healthy individuals. EP Eur..

[35] Nso N., Bookani K.R., Metzl M., Radparvar F. (2021). Role of inflammation in atrial fibrillation: A comprehensive review of current knowledge. J. Arrhythm..

[36] Goette A., Honeycutt C., Langberg J.J. (1996). Electrical Remodeling in Atrial Fibrillation. Circulation.

[37] Souissi W., Bouzid M.A., Farjallah M.A., Ben Mahmoud L., Boudaya M., Engel F.A., Sahnoun Z. (2020). Effect of Different Running Exercise Modalities on Post-Exercise Oxidative Stress Markers in Trained Athletes. Int. J. Environ. Res. Public. Health.

[38] Pedisic Z., Shrestha N., Kovalchik S., Stamatakis E., Liangruenrom N., Grgic J., Titze S., Biddle S.J., E Bauman A., Oja P. (2020). Is running associated with a lower risk of all-cause, cardiovascular and cancer mortality, and is the more the better? A systematic review and meta-analysis. Br. J. Sports Med..

[39] Docherty S., Harley R., McAuley J.J., Crowe L.A.N., Pedret C., Kirwan P.D., Siebert S., Millar N.L. (2022). The effect of exercise on cytokines: Implications for musculoskeletal health: A narrative review. BMC Sports Sci. Med. Rehabil..

[40] Powers S.K., Deminice R., Ozdemir M., Yoshihara T., Bomkamp M.P., Hyatt H. (2020). Exercise-induced oxidative stress: Friend or foe?. J. Sport. Health Sci..

[41] Marini C., De Santis F., Sacco S., Russo T., Olivieri L., Totaro R., Carolei A. (2005). Contribution of Atrial Fibrillation to Incidence and Outcome of Ischemic Stroke. Stroke.

[42] Banerjee A., Taillandier S., Olesen J.B., Lane D.A., Lallemand B., Lip G.Y., Fauchier L. (2013). Pattern of atrial fibrillation and risk of outcomes: The Loire Valley Atrial Fibrillation Project. Int. J. Cardiol..

[43] Murtagh B., Smalling R.W. (2006). Cardioembolic stroke. Curr. Atheroscler. Rep..

[44] Vinding N.E., Kristensen S.L., Rørth R., Butt J.H., Østergaard L., Olesen J.B., Torp-Pedersen C., Gislason G.H., Køber L., Kruuse C. (2022). Ischemic Stroke Severity and Mortality in Patients with and Without Atrial Fibrillation. J. Am. Heart Assoc..

[45] Wolf P.A., Abbott R.D., Kannel W.B. (1991). Atrial fibrillation as an independent risk factor for stroke: The Framingham Study. Stroke.

[46] Kamel H., Okin P.M., Elkind M.S.V., Iadecola C. (2016). Atrial Fibrillation and Mechanisms of Stroke. Stroke.

[47] Kamel H., Healey J.S. (2017). Cardioembolic Stroke. Circ. Res..

[48] Kornej J., Börschel C.S., Benjamin E.J., Schnabel R.B. (2020). Epidemiology of Atrial Fibrillation in the 21st Century. Circ. Res..

[49] Chung S.-C., Sofat R., Acosta-Mena D., A Taylor J., Lambiase P.D., Casas J.P., Providencia R. (2021). Atrial fibrillation epidemiology, disparity and healthcare contacts: A population-wide study of 5.6 million individuals. Lancet Reg. Health-Eur..

[50] She R., Yan Z., Hao Y., Zhang Z., Du Y., Liang Y., Vetrano D.L., Dekker J., Bai B., Lau J.T.F. (2022). Comorbidity in patients with first-ever ischemic stroke: Disease patterns and their associations with cognitive and physical function. Front. Aging Neurosci..

[51] Golüke N.M.S., de Brouwer E.J.M., de Jonghe A., Claus J.J., Staekenborg S.S., Emmelot-Vonk M.H., de Jong P.A., Koek H.L. (2022). Intracranial artery calcifications: Risk factors and association with cardiovascular disease and cognitive function. J. Neuroradiol..

[52] O’neal W.T., Efird J.T., Qureshi W.T., Yeboah J., Alonso A., Heckbert S.R., Nazarian S., Soliman E.Z. (2015). Coronary Artery Calcium Progression and Atrial Fibrillation. Circ. Cardiovasc. Imaging.

[53] Budoff M.J., Young R., Burke G., Carr J.J., Detrano R.C., Folsom A.R., Kronmal R., Lima J.A.C., Liu K.J., McClelland R.L. (2018). Ten-year association of coronary artery calcium with atherosclerotic cardiovascular disease (ASCVD) events: The multi-ethnic study of atherosclerosis (MESA). Eur. Heart J..

[54] Hillerson D., Wool T., Ogunbayo G.O., Sorrell V.L., Leung S.W. (2020). Incidental Coronary Artery Calcification and Stroke Risk in Patients with Atrial Fibrillation. Am. J. Roentgenol..

[55] Wang T.K.M., Chan N., Cremer P.C., Kanj M., Baranowski B., Saliba W., Wazni O.M., A Jaber W. (2021). Incorporating coronary calcification by computed tomography into CHA2DS2-VASc score: Impact on cardiovascular outcomes in patients with atrial fibrillation. EP Eur..

[56] Elsheikh S., Hill A., Irving G., Lip G.Y.H., Abdul-Rahim A.H. (2024). Atrial fibrillation and stroke: State-of-the-art and future directions. Curr. Probl. Cardiol..

[57] Aengevaeren V.L., Mosterd A., Braber T.L., Prakken N.H., Doevendans P.A., Grobbee D.E., Thompson P.D., Eijsvogels T.M., Velthuis B.K. (2017). Relationship Between Lifelong Exercise Volume and Coronary Atherosclerosis in Athletes. Circulation.

[58] De Bosscher R., Dausin C., Claus P., Bogaert J., Dymarkowski S., Goetschalckx K., Ghekiere O., Van De Heyning C.M., Van Herck P., Paelinck B. (2023). Lifelong endurance exercise and its relation with coronary atherosclerosis. Eur. Heart J..

[59] Dores H., de Araújo Gonçalves P., Monge J., Costa R., Tátá L., Cardim N., Neuparth N., Sharma S. (2020). Coronary atherosclerotic burden in veteran male recreational athletes with low to intermediate cardiovascular risk. Rev. Port. Cardiol..

[60] Rogers P.A., Bernard M.L., Madias C., Thihalolipavan S., Mark Estes N.A., Morin D.P. (2018). Current Evidence-Based Understanding of the Epidemiology, Prevention, and Treatment of Atrial Fibrillation. Curr. Probl. Cardiol..

[61] Myrstad M., Berge T., Ihle-Hansen H., Sørensen E., Nystad W., Ranhoff A.H., Aarønæs M. (2020). Stroke in endurance athletes with atrial fibrillation. Eur. J. Prev. Cardiol..

[62] Pallikadavath S.M., Richards C., Bountziouka V., Sandilands A.J., Graham-Brown M.P.M., Robinson T., Singh A., McCann G.P. (2023). The AFLETES Study: Atrial Fibrillation in Veteran Athletes and the Risk of Stroke. Clin. J. Sport. Med..

[63] Svedberg N., Sundström J., James S., Hållmarker U., Hambraeus K., Andersen K. (2019). Long-Term Incidence of Atrial Fibrillation and Stroke Among Cross-Country Skiers. Circulation.

[64] Hållmarker U., Åsberg S., Michaëlsson K., Ärnlöv J., Hellberg D., Lindbäck J., Wester P., James S. (2015). Risk of Recurrent Stroke and Death After First Stroke in Long-Distance Ski Race Participants. J. Am. Heart Assoc..

[65] Van Gelder I.C., Rienstra M., Bunting K.V., Casado-Arroyo R., Caso V., Crijns H.J., De Potter T.J., Dwight J., Guasti L., Hanke T. (2024). 2024 ESC Guidelines for the management of atrial fibrillation developed in collaboration with the European Association for Cardio-Thoracic Surgery (EACTS). Eur. Heart J..

[66] Lampert R., Chung E.H., Ackerman M.J., Arroyo A.R., Darden D., Deo R., Dolan J., Etheridge S.P., Gray B.R., Harmon K.G. (2024). 2024 HRS expert consensus statement on arrhythmias in the athlete: Evaluation, treatment, and return to play. Heart Rhythm.

[67] Akçakoyun M., Alizade E., Gündoğdu R., Bulut M., Tabakcı M.M., Açar G., Avcı A., Şimşek Z., Fidan S., Demir S. (2014). Long-Term Anabolic Androgenic Steroid Use Is Associated with Increased Atrial Electromechanical Delay in Male Bodybuilders. Biomed. Res. Int..

[68] Lincoff A.M., Bhasin S., Flevaris P., Mitchell L.M., Basaria S., Boden W.E., Cunningham G.R., Granger C.B., Khera M., Thompson I.M. (2023). Cardiovascular Safety of Testosterone-Replacement Therapy. N. Engl. J. Med..

[69] Dominic P., Ahmad J., Awwab H., Bhuiyan S., Kevil C.G., Goeders N.E., Murnane K.S., Patterson J.C., Sandau K.E., Gopinathannair R. (2022). Stimulant Drugs of Abuse and Cardiac Arrhythmias. Circ. Arrhythm. Electrophysiol..

[70] Giustetto C., Cerrato N., Gribaudo E., Scrocco C., Castagno D., Richiardi E., Giachino D., Bianchi F., Barbonaglia L., Ferraro A. (2014). Atrial Fibrillation in a Large Population with Brugada Electrocardiographic Pattern: Prevalence, Management, and Correlation with Prognosis. Heart Rhythm.

[71] Johnson J.N., Tester D.J., Perry J., Salisbury B.A., Reed C.R., Ackerman M.J. (2008). Prevalence of Early-Onset Atrial Fibrillation in Congenital Long QT Syndrome. Heart Rhythm.

[72] Pelliccia A., Sharma S., Gati S., Bäck M., Börjesson M., Caselli S., Collet J.-P., Corrado D., Drezner J.A., Halle M. (2021). 2020 ESC Guidelines on sports cardiology and exercise in patients with cardiovascular disease. Eur. Heart J..

[73] Fox K.A.A., E Lucas J., Pieper K.S., Bassand J.-P., Camm A.J., A Fitzmaurice D., Goldhaber S.Z., Goto S., Haas S., Hacke W. (2017). Improved risk stratification of patients with atrial fibrillation: An integrated GARFIELD-AF tool for the prediction of mortality, stroke and bleed in patients with and without anticoagulation. BMJ Open.

[74] Stewart K., Guseh J.-S. (2023). Antithrombotic therapy in athletes: A balancing act. Am. Coll. Cardiol..

[75] Chandran A., Boltz A.J., Morris S.N., Robison H.J., Nedimyer A.K., Collins C.L., Register-Mihalik J.K. (2022). Epidemiology of Concussions in National Collegiate Athletic Association (NCAA) Sports: 2014/15-2018/19. Am. J. Sports Med..

[76] Berkowitz J.N., Moll S. (2017). Athletes and blood clots: Individualized, intermittent anticoagulation management. J. Thromb. Haemost..

[77] Moll S., Berkowitz J.N., Miars C.W. (2018). Elite athletes and anticoagulant therapy: An intermittent dosing strategy. Hematology.

[78] Peigh G., Passman R.S. (2023). “Pill-in-Pocket” anticoagulation for stroke prevention in atrial fibrillation. J. Cardiovasc. Electrophysiol..

[79] Passman R. (2021). “Pill-in-Pocket” Anticoagulation for Atrial Fibrillation: Fiction, Fact, or Foolish?. Circulation.

[80] Reddy V.Y., Doshi S.K., Kar S., Gibson D.N., Price M.J., Huber K., Horton R.P., Buchbinder M., Neuzil P., Gordon N.T. (2017). 5-Year Outcomes After Left Atrial Appendage Closure. J. Am. Coll. Cardiol..

[81] Ko D.T. (2002). β-Blocker Therapy and Symptoms of Depression, Fatigue, and Sexual Dysfunction. JAMA.

[82] https://www.wada-ama.org/en/prohibited-list.

[83] Andrikopoulos G.K. (2015). Flecainide: Current status and perspectives in arrhythmia management. World J. Cardiol..

[84] Kirchhof P., Camm A.J., Goette A., Brandes A., Eckardt L., Elvan A., Fetsch T., van Gelder I.C., Haase D., Haegeli L.M. (2020). Early Rhythm-Control Therapy in Patients with Atrial Fibrillation. N. Engl. J. Med..

[85] Park J., Shim J., Lee J.M., Park J., Heo J., Chang Y., Song T., Kim D., Lee H.A., Yu H.T. (2022). Risks and Benefits of Early Rhythm Control in Patients with Acute Strokes and Atrial Fibrillation: A Multicenter, Prospective, Randomized Study (the RAFAS Trial). J. Am. Heart Assoc..

[86] Han S., Jia R., Cen Z., Guo R., Zhao S., Bai Y., Xie M., Cui K. (2023). Early rhythm control vs. rate control in atrial fibrillation: A systematic review and meta-analysis. Front. Cardiovasc. Med..

[87] Mark D.B., Anstrom K.J., Sheng S., Piccini J.P., Baloch K.N., Monahan K.H., Daniels M.R., Bahnson T.D., Poole J.E., Rosenberg Y. (2019). Effect of Catheter Ablation vs Medical Therapy on Quality of Life Among Patients with Atrial Fibrillation. JAMA.

[88] Johnson B.M., Wazni O.M., Farwati M., Saliba W.I., Santangeli P., Madden R., Bouscher P., Chung M., Kanj M., Dresing T.J. (2023). Atrial Fibrillation Ablation in Young Adults: Measuring Quality of Life Using Patient-Reported Outcomes Over 5 Years. Circ. Arrhythm. Electrophysiol..

[89] Wazni O.M., Dandamudi G., Sood N., Hoyt R., Tyler J., Durrani S., Niebauer M., Makati K., Halperin B., Gauri A. (2021). Cryoballoon Ablation as Initial Therapy for Atrial Fibrillation. N. Engl. J. Med..

[90] Hsu J.C., Darden D., Du C., Marine J.E., Nichols S., Marcus G.M., Natale A., Noseworthy P.A., Selzman K.A., Varosy P. (2023). Initial Findings from the National Cardiovascular Data Registry of Atrial Fibrillation Ablation Procedures. J. Am. Coll. Cardiol..

[91] Akerström F., Hutter J., Charitakis E., Tabrizi F., Asaad F., Bastani H., Bourke T., Braunschweig F., Drca N., Englund A. (2024). Association between catheter ablation of atrial fibrillation and mortality or stroke. Heart.

[92] Prasitlumkum N., Tokavanich N., Siranart N., Techasatian W., Cheungpasitporn W., Navaravong L., Chokesuwattanaskul R. (2023). Atrial fibrillation catheter ablation in endurance athletes: Systematic review and meta-analysis. J. Interv. Card. Electrophysiol..

